# Oxidative modification alters tau condensation and promotes tau-associated cytoplasmic speckle formation

**DOI:** 10.1016/j.jbc.2026.113365

**Published:** 2026-07-24

**Authors:** Shan Sun, Bojun Yang, Siqian Chen, Qiaoting Huang, Yingping Zhang, Ningning Li, Qiang Peng, Kin Yip Tam, Hongfeng Wang, Zheng Ying

**Affiliations:** 1 Children’s Hospital of Soochow University, Jiangsu Key Laboratory of Drug Discovery and Translational Research for Brain Diseases, College of Pharmaceutical Sciences, Soochow University, Suzhou, Jiangsu, China; 2 Faculty of Health Sciences, University of Macau, Taipa, Macau, China; 3 MOE Key Laboratory of Geriatric Diseases and Immunology, College of Pharmaceutical Sciences, Suzhou Medical College of Soochow University, Suzhou, Jiangsu, China; 4 Jiangsu Province Engineering Research Center of Precision Diagnostics and Therapeutics Development, Soochow University, Suzhou, Jiangsu, China

**Keywords:** condensation, phase separation, RNA-binding protein, tau, tau-associated cytoplasmic speckle (TACS)

## Abstract

Oxidative modification-mediated tau aggregation is believed to contribute to multiple aging-related neurodegenerative diseases. Significant evidence indicates that various aging disease-related aggregates are closely linked to biochemical modification-driven abnormal protein phase separation, a thermodynamic conversion to form biomolecular and functional condensates, which are commonly named membrane-less organelles. However, whether oxidative modification regulates tau phase separation remains elusive. In the present study, we show that oxidative modification of cysteine 291 and cysteine 322 in tau impairs the liquidity of tau condensates *in vitro* and in cells, and promotes mis-localization of SRRM2 and SRSF2, the pre-mRNA splicing-related nuclear speckle protein, to cytoplasmic tau aggregates to form tau-associated cytoplasmic speckle (TACS). Interestingly and importantly, we found that RING-Bait technology, which has been shown to selectively target and clear protein aggregates, will be helpful for the elimination of TACS upon oxidative stress exposure. Taken together, our study illustrates the role of oxidative modification in tau phase separation and TACS formation and provides a novel therapeutic strategy for oxidative stress-associated aging-related neurodegenerative diseases.

Tau protein, encoded by the *MAPT* gene, is the major component of intracellular pathogenic inclusions in several aging-related neurodegenerative diseases, especially in neurofibrillary tangles in Alzheimer’s disease (AD) and frontotemporal dementia (FTD) (1). Structurally, tau contains N-terminal region (N1 and N2), proline-rich domain (PRD), and microtubule-binding repeat domains (R1, R2, R3, and R4), and alternative splicing can generate six different isoforms of tau (respectively named 0N3R, 1N3R, 2N3R, 0N4R, 1N4R, and 2N4R, whose classification depends on the splicing status of exons coding N1, N2, and R2), which is developmentally regulated (2). In healthy conditions, tau influences microtubule polymerization as a soluble neuron-specific microtubule-associated protein (3, 4); however, in pathological conditions, tau is misfolded and accumulated to form pathogenic neurofibrillary tangles or other forms of intracellular inclusions (5, 6). Phase separation is a process that forms a reversible dense phase (separated condensates) from a dilute phase by a thermodynamic conversion and condensation, and an aberrant phase separation process is a trigger of neurodegeneration-associated protein aggregation (7, 8, 9). Similar to FUS, TDP-43, and other neurodegenerative disease-associated proteins, tau also undergoes condensation to form tau condensates, and emerging evidence supports that the liquid-to-solid phase transition of tau governs tau fibril formation (2, 10). Although tau condensation plays important roles in biological regulation, the contribution of pathological condensation of tau to AD pathogenesis remains not fully understood.

Several observations suggested that various post-translational modifications (PTMs), which can rapidly and reversibly and spatiotemporally switch protein condensation by modulating tau protein function, regulate tau condensation and aggregate formation (7). For example, phosphorylation and acetylation, two forms of PTMs, respectively promote and disfavor tau condensation to form tau condensates by altering intermolecular electrostatic interaction (11, 12, 13, 14). Oxidative modification, another vital form of PTMs, not only causes the neuro-pathogenic aggregates of tau (15) but also closely relates to abnormal phase separation of aging-related neurodegenerative disease proteins, like amyotrophic lateral sclerosis (ALS)-linked TDP-43 and SOD1 (16, 17). Notably, tau protein contains two oxidative modification-associated residues (cysteine 291 and cysteine 322, hereafter referred to as C291 and C322), which are, respectively, located in R2 and R3 (18). Moreover, previous papers showed that three- and four-repeat (3R and 4R) tau isoforms can be both oxidatively modified, although three-repeat tau isoforms only contain C322 (19, 20). Relevant to this, a crucial question is whether oxidative modification directly regulates tau condensation and, if so, what is the contribution of this regulation to neurodegenerative disease pathogenesis.

There are many biomolecular condensates lacking surrounding membranes, named membrane-less organelles (MLOs), which are formed by multivalent macromolecular interaction-driven condensation and perform the “bioreactor” roles in eukaryotic cells (21). It is clear that cytoplasmic tau aggregates mis-localize multiple components of nuclear speckle, which is a pre-mRNA splicing-related nuclear MLO (22, 23, 24, 25). Moreover, the cytoplasmic distribution of nuclear speckle protein, similar to the distribution of pathogenic tau neurofibrillary tangle deposition in the early stage of AD, is also found in the hippocampus and amygdala region of human AD cases (26). In addition to changing the organization of nuclear speckle components, tau aggregates also alter the property and function of nuclear speckles, including their dynamics and pre-mRNA splicing (23, 27). A recent paper showed that oxidative modification alters the condensation property of splicing factor proline- and glutamine-rich (SFPQ) and then leads to SFPQ re-localization from paraspeckle (nuclear MLO) to the stress granule (cytoplasmic MLO) under stress conditions (28); however, whether oxidative modification involves in the mis-localization of nuclear speckle protein to cytoplasmic tau aggregates in AD remains elusive.

Here, we studied the effect of oxidative modification on tau condensation and aggregation and found that oxidative modification of tau at C291 and C322 decreases the size and liquidity of tau condensates to promote tau fibril formation *in vitro* and in cells. Moreover, oxidative modification of tau triggers aberrant positioning of nuclear speckle proteins into cytoplasmic tau aggregates to form pathological tau-associated cytoplasmic speckle (TACS). Notably, we found that tau-RING, a RING-Bait degrader that is composed of the RING domain of E3 ubiquitin ligase TRIM21 and tau (0N4R) mutation of proline 301 to a serine (P301S) as a Bait, effectively removes oxidative modification-associated pathological TACS to ameliorate tau aggregation-mediated neurodegeneration.

## Results

### Tau undergoes RNA-associated phase separation *in vitro*, which is affected by oxidative stress in association with C291 and C322 residues

**Figure 1:**
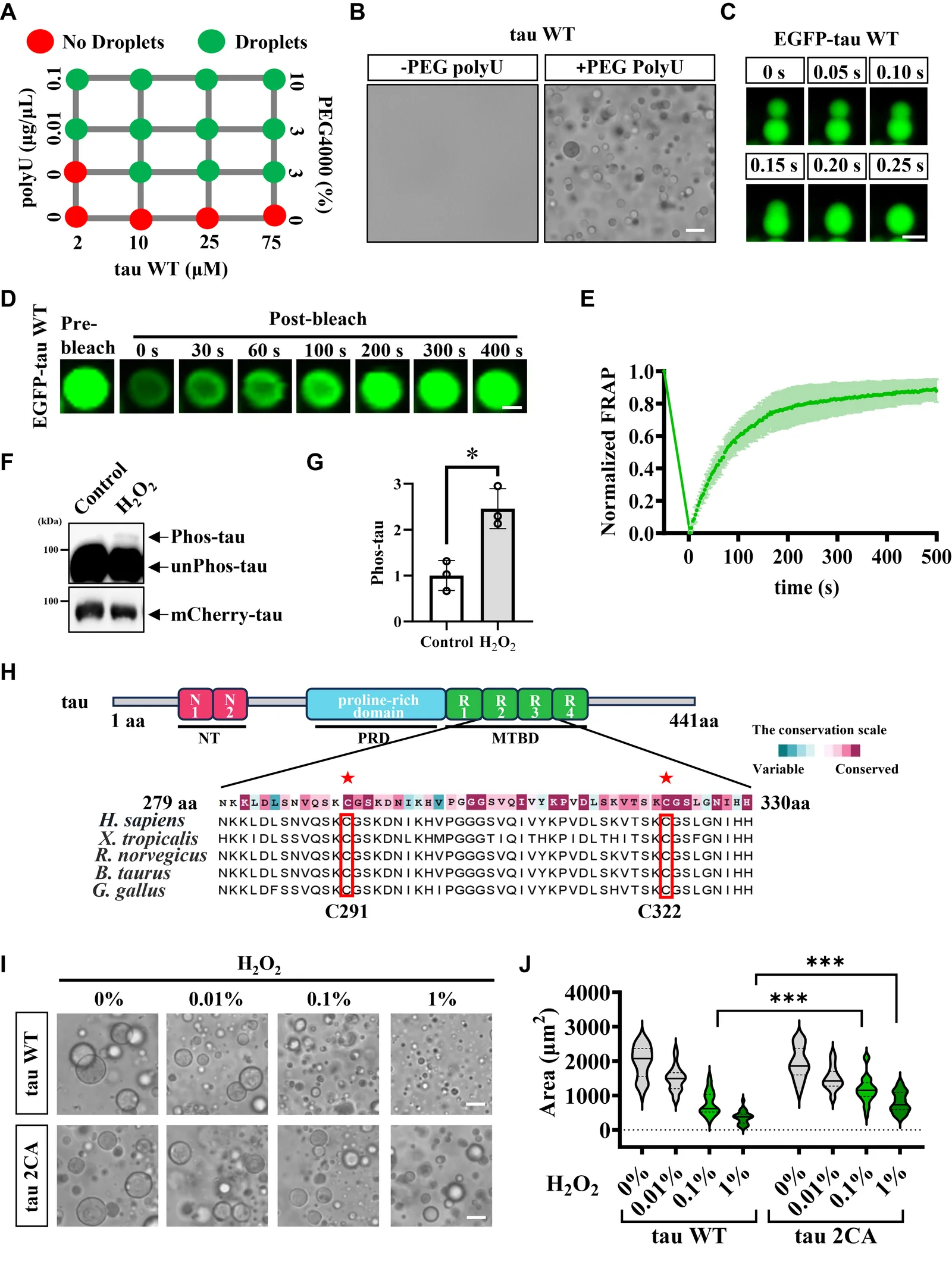
**Oxidative modification of C291 and C322 residues alters tau phase separation behavior.***A*, summary of unlabeled tau phase separation behaviors *in vitro* with increasing concentrations of tau, PEG4000, and polyU. *B*, phase separation of unlabeled tau (25 μM) with or without molecular crowding agent (10% PEG4000) and polyU (0.01 μg/μl) with 150 mM NaCl *in vitro*. Scale bar, 10 μm. *C*, the fusion event of EGFP-tau condensates (25 μM tau, 10% PEG4000, 0.01 μg/μl polyU, and 150 mM NaCl) *in vitro*. Scale bar, 5 μm. *D*, representative image of FRAP analysis of EGFP-tau condensate (25 μM tau, 10% PEG4000, 0.01 μg/μl polyU, and 150 mM NaCl) *in vitro*. Scale bar, 1 μm. *E*, quantification of fluorescence intensity of EGFP-tau condensates in (*D*). Quantification data were shown as mean ± S.D. from three independent experiments. *F*, HEK293 cells expressing mCherry-tau WT were treated with 200 μM H_2_O_2_ for 15 min. The cell lysates were analyzed by Phos-tag gels to show phosphorylated tau (Phos-tau) and unphosphorylated tau (unPhos-tau) with the indicated antibodies. *G*, quantification of Phos-tau in (*F*). Data are shown as mean ± S.D. from three independent experiments. ∗*p* < 0.01, paired *t* test. *H*, the sequence alignment of tau homologs across different species. *I*, phase separation of unlabeled tau (25 μM tau WT or 2CA, 10% PEG4000, 0.01 μg/μl polyU, and 150 mM NaCl) with increasing concentrations of H_2_O_2_. Scale bar, 10 μm. *J*, quantification of tau condensate size in (*I*) from three independent experiments. The black line represents the median, and the black dotted line represents the quartile. ∗∗∗*p* < 0.001, two-way ANOVA followed by *post hoc* Tukey’s tests.

Given that tau performs condensation by phase separation *in vitro* and *in vivo* (10, 13, 29, 30, 31), we examined whether tau undergoes phase separation at physiological salt concentration (150 mM NaCl) *in vitro*. Notably, we didn’t observe tau phase separation at a physiological level of tau in neurons (∼2 μM) without RNA (32) *in vitro* (Fig. 1*A*). However, tau did exhibit concentration-dependent phase separation upon addition of long and low complexity RNA (polyuridylic acid, polyU) and the molecular crowding agent (polyethylene glycol, PEG) (Fig. 1, *A* and *B*). These data indicate that tau phase separation at physiological concentrations depends on RNA and molecular crowding conditions, similar to the previous study reported (13, 33), and the tau protein displayed phase separation behavior at 2 μM and higher concentration (25 μM, which drives stronger phase separation behavior) (Fig. S1*A*). To study the dynamics and liquidity of tau droplets, we established EGFP-fused tau to observe the droplet fusion and perform a fluorescence recovery after photobleaching (FRAP) experiment. The results showed that EGFP-tau condensates readily fused (Fig. 1*C*) and exhibited rapid recovery after bleaching in the FRAP experiment, indicating the dynamic exchange of EGFP-tau between the dense phase and the surrounding dilute phase (Fig. 1, *D* and *E*). Taken together, these findings suggest that phase-separated tau condensates, which are formed under RNA and crowding conditions, display key characteristics of liquid property *in vitro*.

Oxidative stress has been reported to be closely linked to phase separation-mediated abnormal aggregation of aging-related neurodegenerative disease proteins (34), such as amyotrophic lateral sclerosis (ALS)-linked TDP-43 and SOD1 (16, 17). To explore the potential contribution of oxidative stress to tau aggregation-associated hyperphosphorylation, we used Phos-tag gels to perform biochemical analysis and found that oxidative stress could enhance tau phosphorylation in cells (Fig. 1, *F* and *G*). To further study the potential of oxidative modification mediated by oxidative stress on tau phase separation, we performed the sequence alignment of tau protein homologs in different species, and identified two highly conserved cysteine residues, C291 and C322 (Fig. 1*H*), which have been showed to be associated with the assemble of paired helical filaments by regulating redox potential and the fibrillization of tau, and may influence the ability of phase separation of tau (18, 35). Next, we performed site-directed mutagenesis of cysteine to alanine (C291A/C322A, hereafter referred to as 2CA) and found that the treatments of hydrogen peroxide (H_2_O_2_) (0.01%-1%) with increasing concentration reduced the size of tau wild type (WT) condensates, but tau 2CA was relatively resistant to this treatment (Fig. 1, *I* and *J*). Moreover, tau condensates displayed lower liquidity and less dynamics upon H_2_O_2_ treatments with increased concentration (data not shown). These results indicate that ROS-driven oxidative modification of tau at C291 and C322 residues alters its phase separation behavior.

### Oxidative modification of tau drives pathogenic fibril formation by aberrant phase transition

**Figure 2:**
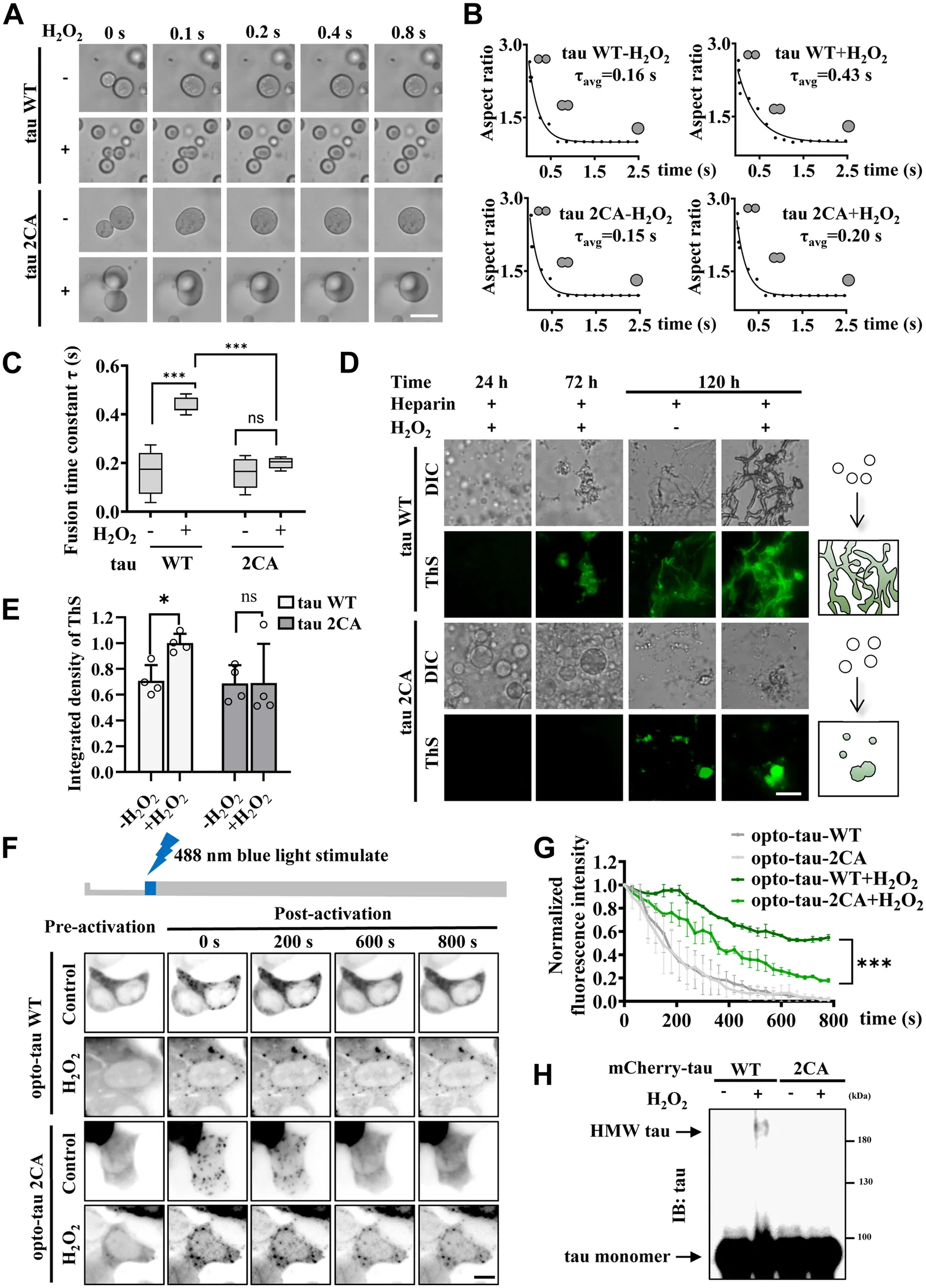
**The liquidity of tau condensates is impaired by oxidative modification.***A*, the fusion event of tau condensates (25 μM tau, 10% PEG4000, 0.01 μg/μl polyU, 0.1% H_2_O_2,_ and 150 mM NaCl) *in vitro*. Scale bar, 8 μm. (*B*-*C*) Aspect ratio as a function of time in (*A*) was fit to an exponential equation of decay (*B*) to determine relaxation time τ (*C*). Boxplot (min-max) of relaxation time τ for experiments in (*C*). ∗∗∗*p* < 0.001; ns, not significantly different, two-way ANOVA followed by *post hoc* Tukey’s tests. *D*, the formation of tau WT or 2CA fibrils (25 μM tau WT or 2CA, 10% PEG4000, 0.01 μg/μl polyU, 0.1% H_2_O_2_, and 150 mM NaCl) induced by heparin (10 μM) under the indicated times *in vitro*. The fibrils of tau WT or 2CA were stained by ThS. Scale bar, 10 μm. *E*, the integrated ThS fluorescence intensity of tau WT or 2CA fibrils at 120 h in (D). Data were shown as mean ± S.D. from three independent experiments. ∗*p* < 0.05; ns, not significantly different, unpaired *t* test. *F*, HEK293 cells expressing opto-tau (WT or 2CA) with or without H_2_O_2_ treatment (200 μM, 15 min) were stimulated with a single *blue light* pulse, and time-lapse imaging was used to record the reversibility of tau condensates. Scale bar, 10 μm. *G*, the integrated fluorescence intensity of opto-tau (WT or 2CA) in the dense phase (condensates) in (*F*). Data are shown as mean ± S. D. The statistical analysis was normalized fluorescence intensity corresponding to the final time point; ∗∗∗*p* < 0.001, two-way ANOVA followed by *post hoc* Tukey’s tests. *H*, HEK293 cells expressing mCherry-tau (WT or 2CA) were treated with 200 μM H_2_O_2_ for 15 min. The cell lysates were analyzed by a non-reducing protein immunoblot experiment with the indicated antibodies.

To further study the effect of oxidative modification on tau droplet dynamics, we performed the droplet fusion assay and a statistical assay for the fusion timescale (as indicated by relaxation time τ_avg_) (34, 36). The results showed that H_2_O_2_-induced oxidative modification decreased droplet fusion dynamics of tau WT, but not tau 2CA (Fig. 2, *A*–*C*). To assess the contribution of oxidative stress to pathogenic tau fibrillization, we used *in vitro* polyanionic co-factors RNA and heparin, which are widely used co-factors in tau fibrillization (14, 35, 37). The Thioflavine S (ThS) staining results showed that H_2_O_2_-induced oxidative modification increased the fibrillization speed of tau WT *in vitro*, but not tau 2CA (Fig. 2, *D* and *E*).

We further studied the effect of oxidative stress on tau condensation in cells using a light-inducible “OptoDroplet” system (38), in which we respectively fused tau WT and 2CA with blue light (488 nm)-sensitive and self-oligomerization domain (Cry2_PHR_) to establish light-inducible “opto-tau WT” and “opto-tau 2CA” systems. Given that tau co-condenses with microtubules in cells (39, 40, 41), we first disrupted the microtubule network using nocodazole and found that tau WT and 2CA both underwent condensation after blue light stimulation (Fig. 2, *F* and *G*). However, H_2_O_2_-treatment delayed the disassembly of tau WT condensates (after blue light withdrawal), but tau 2CA was relatively resistant to this treatment (Fig. 2, *F* and *G*). Moreover, we studied the effect of H_2_O_2_-induced oxidative modification on the formation of reducing- and heat-stable high molecular weight (HMW) tau multimer, a typical characteristic of pathogenic tau multimerization. As shown in our results, the treatment of H_2_O_2_ promoted the formation of HMW tau WT multimer, but not HMW tau 2CA multimer (Fig. 2*H*). Taken together, these data suggest that oxidative modification of tau triggered by H_2_O_2_-mediated oxidative stress promotes pathology-related tau phosphorylation and further multimerization.

### Oxidative stress promotes TACS assembly by oxidation of tau at C291 and C322

**Figure 3:**
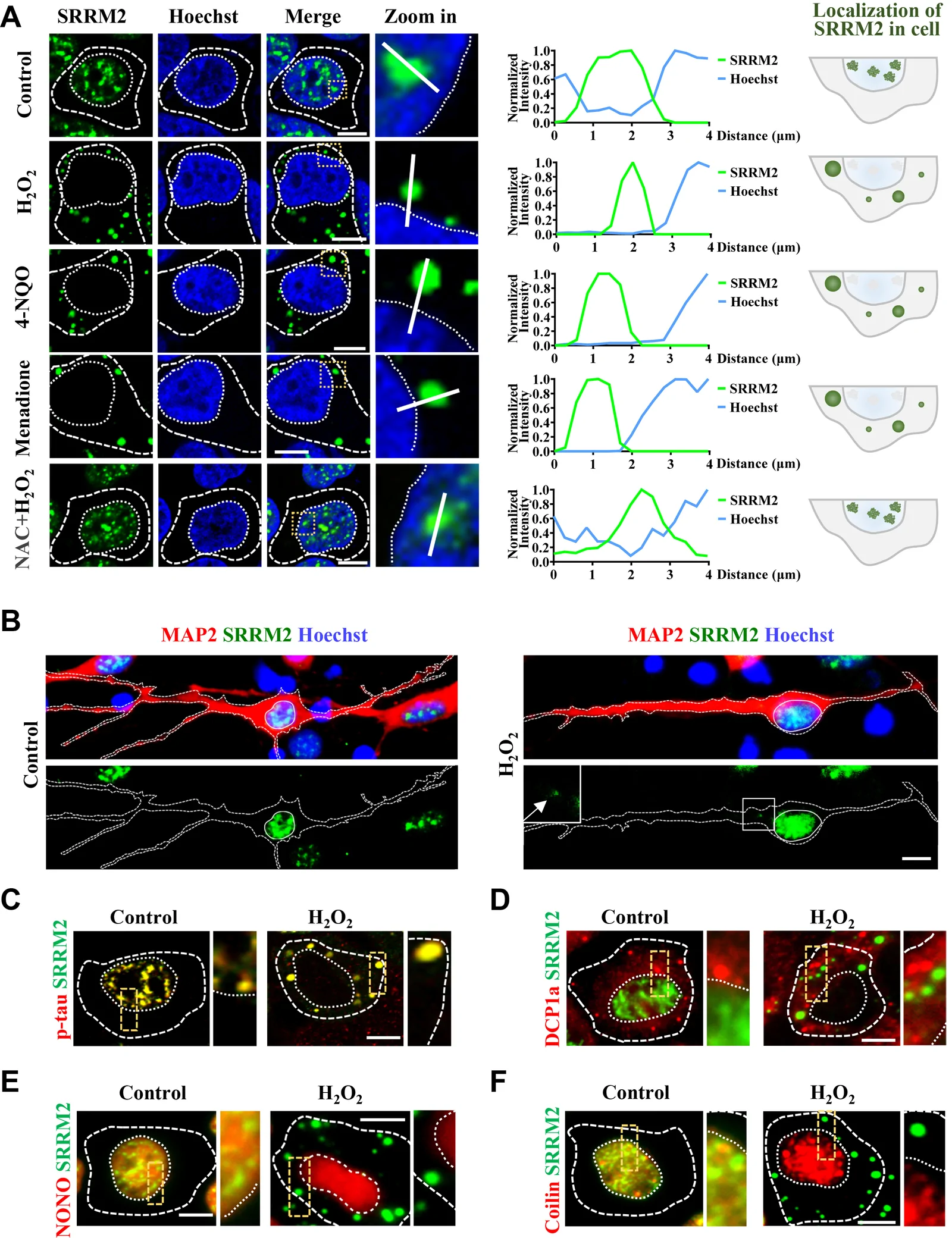
**Oxidative stress triggers TACS formation.***A*, HEK 293 cells were treated with H_2_O_2_ (200 μM, 15 min), 4-NQO (5 μg/μl, 30 min), Menadione (0.5 mM, 30 min), or a combination of NAC (5 mM, pretreated for 20 min) and H_2_O_2_ (200 μM, 15 min). Then the cells were fixed and stained with anti-SC-35 antibody and Hoechst. Scale bar, 10 μm. The line scan shows the fluorescent intensity of nuclear speckle protein and Hoechst. *B*, primary cultured mouse cortical neurons were treated with or without H_2_O_2_ (200 μM, 15 min), and then were fixed and stained with anti-MAP2, anti-SC-35 antibodies and Hoechst. Scale bar, 10 μm. *C-F*, HEK 293 cells were treated with or without H_2_O_2_ (200 μM, 15 min), and then fixed and stained with anti-SC-35 and p-tau (T231) (*C*), or anti-DCP1a (P body marker) (*D*), or anti-NONO (paraspeckle marker) (*E*), or anti-Coilin (Cajal body marker) (*F*) antibodies. Scale bar, 10 μm.

The evidence showed that nuclear speckle proteins mislocalize to cytoplasmic tau aggregates in the brains of AD and FTD patients (23, 26), and we named this new MLO containing tau and nuclear speckle proteins as TACS. In our study, we tested whether oxidative stress could induce TACS formation. To this end, we used various reactive oxygen species (ROS) generators, including H_2_O_2_, menadione, and 4-Nitroquinoline N-oxide (4-NQO). We found that all these ROS generators could induce cytoplasmic mis-translocation of nuclear speckle protein by immunofluorescence staining using anti-SC-35 (SRSF2) antibody, which has been shown to mainly target nuclear speckle protein serine arginine repetitive matrix protein 2 (SRRM2) (Fig. 3*A*) (42). In contrast, antioxidant N-acetyl cysteine (NAC) rescued the H_2_O_2_-induced cytoplasmic translocation of nuclear speckle proteins (Fig. 3*A*). To further verify this phenomenon in neurons, we treated mouse primary cortical neurons with H_2_O_2_ and also found nuclear speckle proteins translocated to the cytoplasm and formed condensates (Fig. 3*B*). Moreover, we found phosphorylated tau at T231, a crucial phosphorylation site in tau pathogenic aggregation (43, 44), also colocalized with these nuclear speckle protein-associated cytoplasmic condensates (Fig. 3*C*), indicating these condensates of cytoplasmic nuclear speckle protein are TACS. These results suggest that ROS promotes nuclear speckle protein mislocalization to cytoplasmic tau aggregates to form cytoplasmic TACS. We further studied the association between TACS and other MLOs and found that TACS is a kind of new MLO that differs from other MLOs (DCP1a-marked processing bodies, NONO-marked paraspeckles, Coilin-marked Cajal bodies, or G3BP1-marked stress granules) (Fig. 3, *D*–*F*, and S2*A*). These data suggest that oxidative stress promotes the formation of TACS, a new MLO in the cytoplasm.

**Figure 4:**
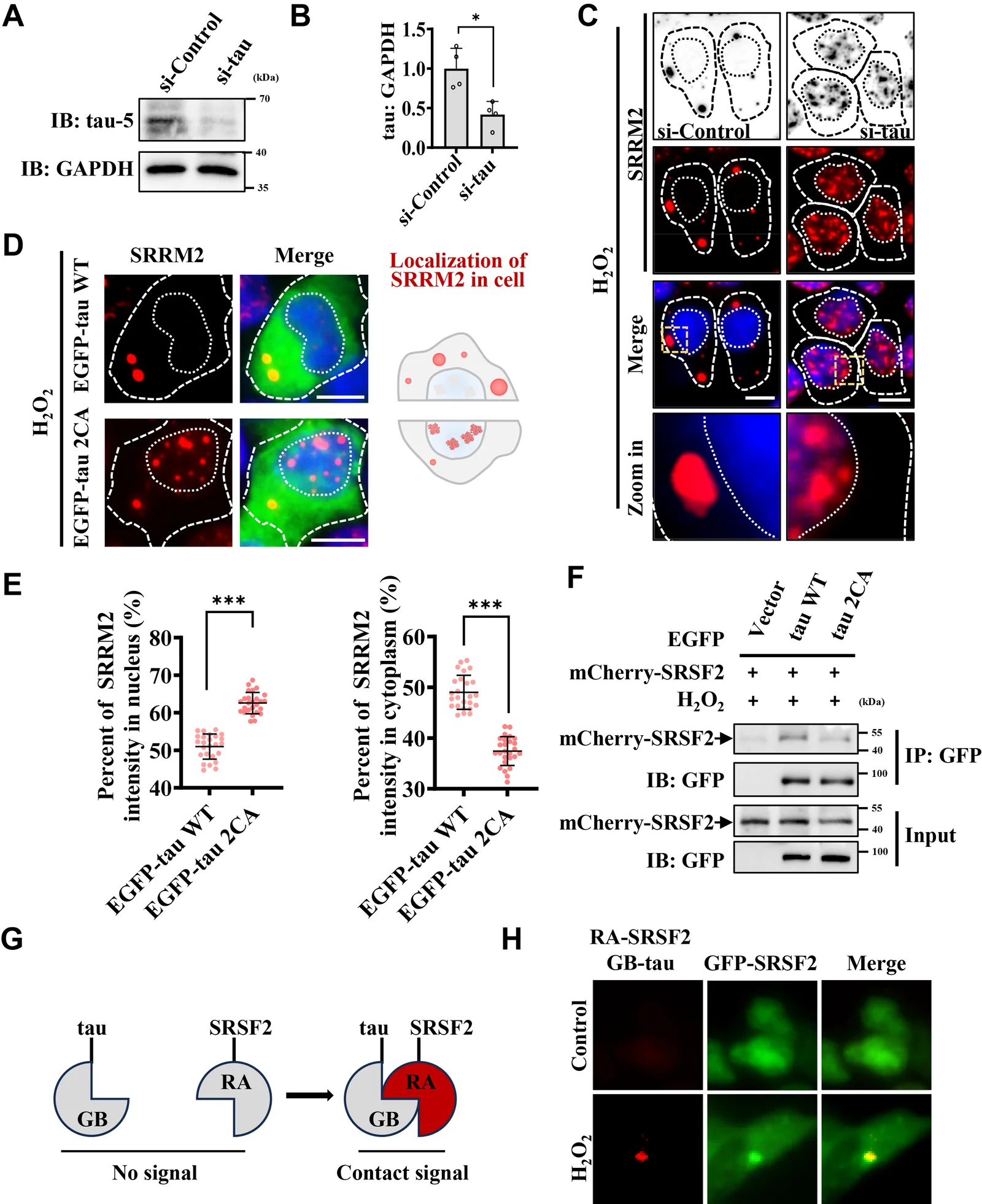
**Contribution of tau oxidative modification to TACS formation.***A*, HEK 293 cells were transfected with control or tau siRNA for 36 h, and then the cells were analyzed by immunoblot with the indicated antibodies. *B*, quantification of the tau protein levels in (*A*). Data are shown as mean ± S.D. from four independent experiments. ∗*p* < 0.05, paired *t* test. *C*, HEK 293 cells were transfected with control or tau siRNA, and then were treated with H_2_O_2_ (200 μM, 15 min). The cells were fixed and stained with anti-SC-35 antibody and Hoechst. Scale bar, 10 μm. *D*, HEK 293 cells were transfected with tau siRNA, followed by transfection with EGFP-tau WT or 2CA, and then were treated with H_2_O_2_ (200 μM, 15 min). The cells were fixed and stained with anti-SC-35 antibody and Hoechst. Scale bar, 10 μm. *E*, quantification of the ratio of nuclear speckle protein intensity in the nucleus or cytoplasm in (*D*). Data are shown as mean ± S.D., n ≥ 26. ∗∗∗*p* < 0.001, unpaired *t* test. *F*, HEK 293 cells expressing mCherry-SRSF2 with EGFP-vector, EGFP-tau WT, or EGFP-tau 2CA, were treated with 200 μM H_2_O_2_ for 15 min. The cell lysates were subjected to immunoprecipitation analysis using anti-GFP antibody and then were analyzed by immunoblot with the indicated antibodies. *G*, the schematic diagram of the dimerization-dependent fluorescent protein system used to assay SRSF2 (RA-SRSF2) and tau (GB-tau) contact. *H*, HEK 293 cells were transfected with RA-SRSF2, GB-tau, and GFP-SRSF2, and then were treated with or without 200 μM H_2_O_2_ for 15 min. Scale bar, 10 μm.

To explore the role of tau in TACS formation, we performed a tau depletion experiment (Fig. 4, *A* and *B*) and found that nuclear speckle protein SRRM2 remained in the nucleus in tau-depleted cells after H_2_O_2_ treatment (Fig. 4*C*), indicating that tau protein is an important reason for the cytoplasmic translocation of SRRM2. As we have shown that oxidative stress not only increased oxidative modification of tau but also promoted TACS assemblies, we wondered whether oxidative stress-mediated TACS formation is regulated by tau oxidative modification. The results showed that, in contrast to cells expressing tau WT, a proportion of SRRM2 failed to translocate to the cytoplasm in cells expressing tau 2CA after H_2_O_2_ treatment for the same time (Fig. 4, *D* and *E*). To further study the effect of tau oxidative modification on the association of tau with another nuclear speckle protein SRSF2, we studied the tau-SRSF2 interaction by performing a co-immunoprecipitation experiment and found that 2CA mutant tau decreased the tau-SRSF2 interaction (Fig. 4*F*). Moreover, we fused SRSF2 with the RA (red fluorescence–capable) domain and fused tau with the GB (core) domain to establish a dimerization-dependent fluorescent protein system in which red fluorescence reflects those two tag-fused proteins are close enough for dimerization (Fig. 4*G*) and found that tau contacted with SRSF2 in the cytoplasm after H_2_O_2_ treatment (Fig. 4*H*). Together, these results suggest that oxidative stress-driven oxidation of tau C291 and C322 residues plays a crucial role in TACS assemblies.

### RING-bait strategy for intracellular TACS clearance

**Figure 5:**
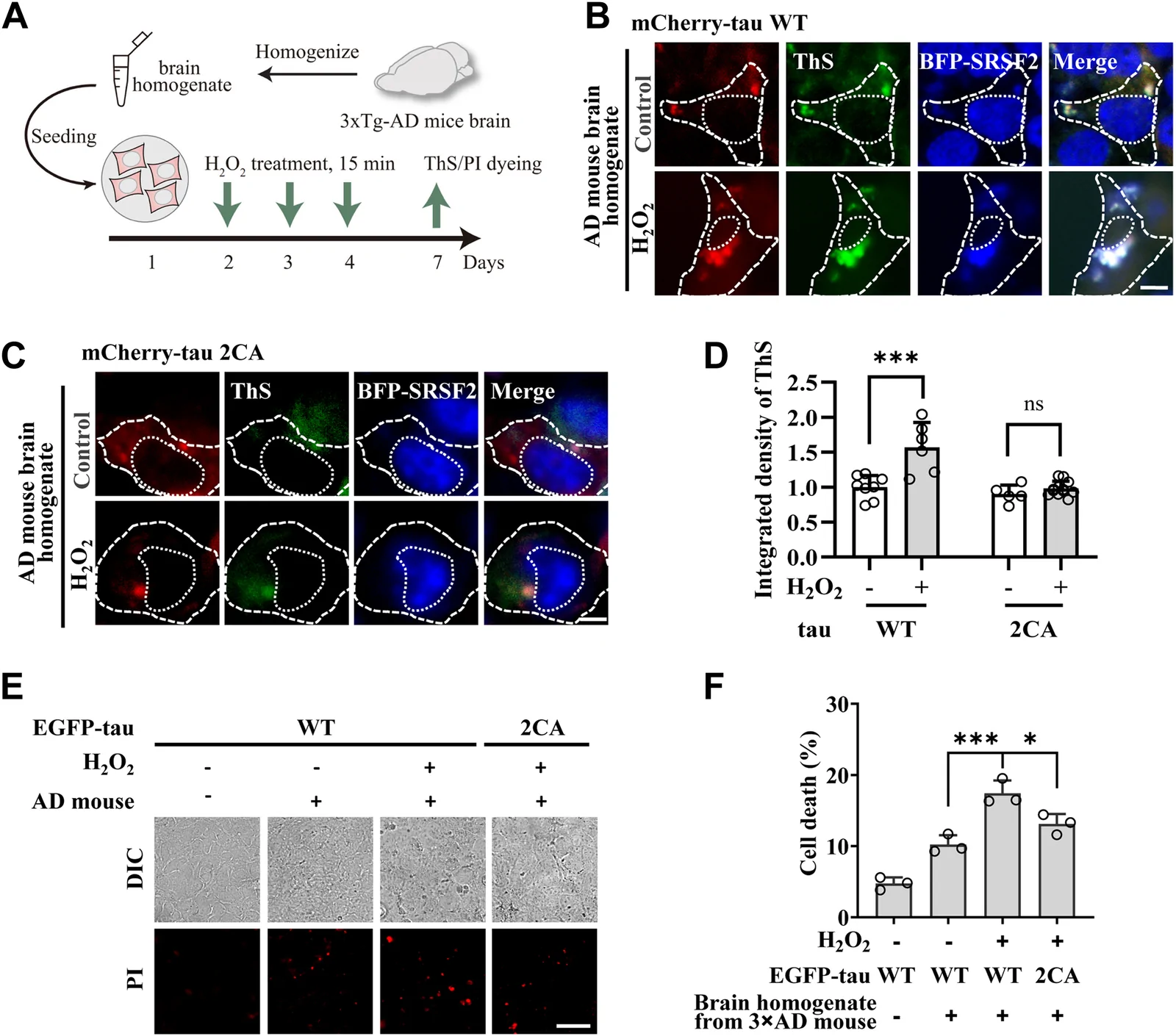
**Oxidative modification of tau triggers TACS-mediated cell death.***A*, the schematic diagram of experiments of aggregate seeding and oxidative stress in cells. *B-C*, HEK 293 cells expressing BFP-SRSF2 and mCherry-tau WT (*B*) or mCherry-tau 2CA (*C*) were treated as shown in (*A*) and then stained by ThS. Scale bar, 10 μm. *D*, the integrated ThS fluorescence intensity of tau WT (*B*) or 2CA (*C*) fibrils. Data were shown as mean ± S.D., n ≥ 5. ∗∗∗*p* < 0. 001; ns, not significantly different, two-way ANOVA followed by *post hoc* Tukey’s tests. *E*, HEK 293 cells expressing EGFP-tau WT or 2CA were treated as (*A*) and then stained with PI. Scale bar, 40 μm. *F*, The integrated PI fluorescence intensity of tau WT or 2CA fibrils in (*E*). Data were shown as mean ± S.D. from three independent experiments. ∗*p* < 0.05, ∗∗∗*p* < 0.001, one-way ANOVA (Compare the mean of each column with the mean of every other column) followed by *post hoc* Tukey’s tests.

To decipher the effect of tau oxidative modification-mediated SRSF2 cytoplasmic translocation on AD pathological process, we treated the tau aggregate seeding cell model with intervals of H_2_O_2_ treatment to mimic chronic and intermittent AD pathogenic process in live cells (Fig. 5*A*) and found that H_2_O_2_ extraordinarily promoted the cytoplasmic translocation of SRSF2 and the seeding speed of aggregates in tau WT-expressing cells, but not in tau 2CA-expressing cells (Fig. 5, *B*–*D*). We further distinguished the cell viability in the seeding cell model using propidium iodide (PI) and found that the cell viability was inhibited after tau aggregate seeding treatment. Specifically, we found that the treatment of H_2_O_2_ further aggravated tau aggregate-induced cell death, which was inhibited by expressing tau oxidative modification inactivated mutant (Fig. 5, *E* and *F*).

**Figure 6:**
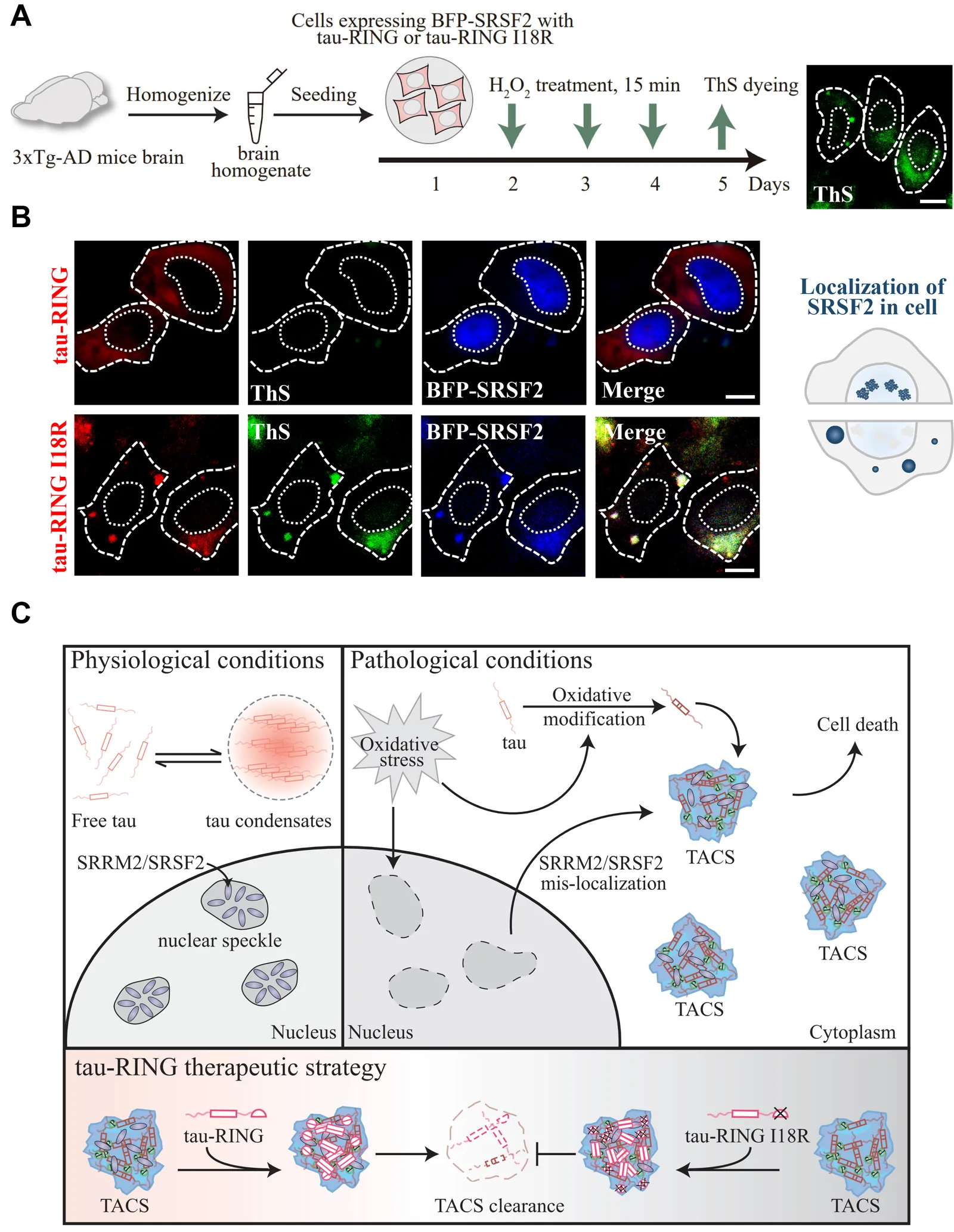
**RING-Bait degrader effectively removes oxidative stress-induced TACS.***A*, the schematic diagram of experiments of aggregate seeding and oxidative stress in cells. *B*, HEK 293 cells expressing BFP-SRSF2 with mCherry-tau-RING or mCherry-tau-RING I18R were treated as shown in (*A*) and then stained by ThS. Scale bar, 10 μm. *C*, schematic model illustrating that tau oxidative modification modulates TACS formation. Under normal conditions, tau undergoes reversible phase separation, and SRRM2 and SRSF2 localize in nuclear speckles. In case of neurodegenerative disease, oxidative stress induces oxidative modification of tau, triggering tau aggregation, which promotes SRRM2 and SRSF2 localization from nuclear speckle to cytoplasmic TACS and results in cell death. In addition, tau-RING degrader, but not tau-RING I18R, can effectively remove abnormal TACS.

According to a previous study, tau-RING, an effective RING-Bait degrader, specifically removes tau aggregates to reduce tau pathology in AD (45). In brief, tau-RING consists of tau aggregate-targeting 0N4R tau P301S as the “Bait” and the RING domain of E3 ligase TRIM21. To study the effect of tau-RING on oxidative stress-driven TACS aggregation, we expressed it in a tau aggregate seeding cell model (Fig. 6*A*). The results showed that tau-RING degrader efficiently removed aggregates, and SRSF2 was still localized in nuclear speckle in tau-RING-expressing cells after tau aggregate seeding and H_2_O_2_ treatment (Fig. 6*B*). However, tau-RING I18R mutant, which was previously shown to prevent its activities (46), failed to prevent tau cytoplasmic translocation and degrade TACS aggregates (Fig. 6, *A* and *B*). Taken together, these data suggest that RING-Bait technology is an effective tool in preventing pathological MLO TACS (Fig. 6*C*).

## Discussion

Oxidative stress is a key factor in neuro-pathogenic aggregate formation in many aging-related neurodegenerative diseases, and oxidative stress-related oxidative modification plays critical roles in the regulation of physiological protein condensation and pathological protein aggregation (15, 19, 47). For example, oxidation of TIA1, a nucleating-associated protein of cytoplasmic MLO stress granule, has been shown to inhibit stress granule formation, a classic cytoplasmic MLO linked to neurodegenerative disease (48). In contrast, disulfide cross-linked species between oxidative cysteines have previously been found in the brain of patients with TDP-43 aggregates (49), and cysteine oxidation has recently been identified as the driving force of the liquid-to-solid transition of TDP-43 (50). Relevant to this, we here show the effect of the oxidative modification on tau-associated MLO formation by intervening in tau phase behavior, which may finally trigger neurodegenerative disease progression (Figs. 1 and 2).

The hyperphosphorylation of tau is a prominent event disrupting its physiological function and leading to pathological aggregation in AD and other neurodegenerative diseases. Oxidative stress, the critical early event of AD pathogenesis, is believed to be implicated in tau abnormal hyperphosphorylation and pathological aggregation, but this hypothesis of a two-step regulation of tau pathogenesis lacks formal demonstration. The present study sheds light on the effect of tau oxidation as an early event that promotes subsequent tau phosphorylation (Fig. 1*F*). Given that the two-step regulation (from oxidative stress to phosphorylation) of ALS-linked protein SOD1 is determined by altering its interaction with protein kinase (51), we speculate that oxidative modification of tau may alter its phosphorylation by affecting its interaction with phosphatase or kinase, thereby triggering tau aggregation and disease progression of AD.

Previous studies showed that pre-mRNA splicing is disturbed in patients with tau-associated neurodegenerative diseases, including AD (52, 53). Similarly, we observed that oxidative modification of tau regulates mis-localization of pre-mRNA splicing-related nuclear speckle protein to cytoplasm (Figs. 3 and 4). We speculate that spatial redistribution of nuclear speckle protein may cause global remodeling of pre-mRNA splicing, including abnormal splicing of tau pre-mRNA (such as the inclusion rate of exon 10, which encodes tau R2 domain) (44), thereby generating more aggregation-prone tau isoforms. Moreover, the expression of different tau isoforms is developmentally regulated (2). For example, health adult cerebral cortex expresses all six tau isoforms, but human fetal brain mainly expresses tau 0N3R isoform (54). Correlatively, three-repeat tau isoforms only contain oxidative modification-associated C322, but not C291 (18, 19). Therefore, it is possible that the developmental shift in the expression of tau isoforms may affect the effect of oxidative stress on tau, and it will be interesting to explore oxidative modification-regulated tau condensation during development in the future.

Interestingly, the fibril-like TACS has also been observed in the brains of AD and FTD patients with tau aggregation (23, 26). A key question is the biological function of oxidative stress-driven TACS containing nuclear speckle protein mislocalization. In the present study, we observed that tau forms fibril-like aggregates with nuclear speckle protein, triggering cell death in the tau aggregate seeding model under oxidative stress (Fig. 5).

Importantly, vital roles have been found for RING-Bait technology, which has been demonstrated to effectively target and remove tau aggregates, but whether the RING-Bait degrader can target aberrant and unique MLO-containing tau, such as TACS, remains unclear. In Figure 6, we show that the RING-Bait degrader effectively targets and removes TACS in cells. Compared with traditional antibody-associated treatment methods, the tau-RING degrader utilizes the natural and specific protein recognition ability of the “bait” to achieve selective capture of dynamic and transient tau intermediate states, thereby enhancing the detection sensitivity and spatiotemporal resolution for pathological tau. Future work is needed to determine the treatment effect of RING-Bait technology on abnormal condensation of tau *in vivo*.

We conclude the effect of oxidative stress-mediated oxidative modification on tau condensation and TACS formation and offer a promising therapeutic avenue targeting neurodegeneration-related TACS.

## Experimental procedures

### Plasmid constructs and siRNAs

EGFP-Vector, His-EGFP-tau WT, EGFP-tau, opto-tau, and EGFP-SRSF2 were described previously (55, 56, 57). mCherry-tau WT was generated by homologous recombination using ClonExpress II One Step Cloning Kit (Vazyme) with the following primers: 5′-AGATCCGCTAGCGCCACCGGCAGCGGCAGCCCACCG-3′ and 5′-GGTGGCGCTAGCGGATCT-3′ from opto-tau WT. His-tau WT was generated by homologous recombination from His-mCherry-tau with the following primers: 5′-AGCCATATGGTCGACGCGGCCGCTTCTCGAGAT-3′ and 5′-CGCGTCGACCATATGGCTGCCGCGCGGCACCAG-3'. His-tau 2CA (C291A, C322A), EGFP-tau 2CA (C291A, C322A), mCherry-tau 2CA (C291A, C322A) and opto-tau 2CA (C291A, C322A) were respectively generated by homologous recombination with following primers: 5′-GCCGGCTCAAAGGATAATATCAAACACGTCCC-3′ and 5′-TTATCCTTTGAGCCGGCCTTGGACTGGACGTTGCTAAGAT-3′ for C291A with 5′-TCCAAGGCCGGCTCATTAGGCAACATCCATCA-3′ and 5′-AATGAGCCGGCCTTGGAGGTCACCTTGCTCAGG-3′ for C322A from His-tau WT, EGFP-tau WT, mCherry-tau WT and opto-tau WT. The RING domain of Trim21 was obtained by cloning PCR product from a human fetal brain cDNA library, and mCherry-tagged tau-RING (P301S) was generated by homologous recombination with the following primers: 5′-TGGATCCGAATGGCTTCAGCAGCACGCTTGACA-3′, 5′-TGAAGCCATTCGGATCCACAAACCCTGCTTGGC-3′, 5′-AGGAGGCCAGAGAGTATCGCGGCCGCACCGGAT-3′, and 5′-GATACTCTCTGGCCTCCTGGCTGATTTCTTTAA-3'. mCherry-tau-RING (I18R) was generated by homologous recombination with following primers: 5′-ACATGCCCTCGTTGCCTGGACCCCTTCGTGGA-3′ and 5′-AGGCAACGAGGGCATGTGACCTCCTCCCACAT-3′ from mCherry-tau-RING. mCherry-SRSF2 was created by subcloning PCR product from EGFP-SRSF2 into mCherry vector with EcoRI/BamHI sites. BFP-SRSF2 was created by subcloning PCR product from EGFP-SRSF2 into BFP vector with XhoI/BamHI sites. RA-SRSF2 was created by subcloning PCR product from EGFP-SRSF2 into RA vector with XhoI/BamHI sites. GB-tau was created by subcloning PCR product from His-mCherry-tau into GB vector with SalI/BamHI sites.

The following siRNAs (GenePharma) were used: Human tau: 5′-CAUGUCCUACGUGAAGGAUGATT-3′, and Negative control: A non-targeting oligonucleotide.

### Protein purification *in vitro*

Protein purification was performed as we previously described (55). Briefly, His-EGFP-tau WT, His-tau WT, and His-tau 2CA (C291A, C322A) proteins were respectively expressed at 16 °C overnight (induced by IPTG) and then purified using Ni Sepharose FF (Vazyme) from BL21 *E.coli* cells. The His tag in these proteins was cleaved using thrombin (Solarbio), and the proteins were further condensed using Amicon Ultra-0.5 (Merck). These purified proteins were finally stored at −80°C in the stock buffer. Under the given salt concentration and molecular crowding conditions, the purified proteins could undergo condensation to form liquid droplets, and these results were visualized by a microscope. The following molecular crowding materials were used for tau phase separation *in vitro*: poly(U) (Sigma, p9528) and PEG4000 (BBI Life Science).

### Cell culture, transfection, and drug treatment

Human embryonic kidney 293 (HEK 293) cells were cultured in 10% fetal bovine serum (FBS) (Gibco, 10099141C), 90% Dulbecco modified essential medium (DMEM) (Gibco, 11995500), 100 U/ml penicillin, and 100 μg/ml streptomycin (Gibco, 15140122).

The transfection experiment was performed as we previously described (56, 57). Briefly, the indicated plasmids, which were wrapped using Lipofectamine 2000 reagent (Invitrogen, 11668019), were transfected into the cells in Opti-MEM (Gibco, 31985070) (without serum medium). The culture medium was added to these cells after 4 h, and they were treated with the drug or observed after transfection for 24 h. The siRNA, which was wrapped by RNAiMAX transfection reagent (Invitrogen), was transfected into the cells in Opti-MEM according to the manufacturer’s instructions. The following chemicals were used for treatment: nocodazole (Sigma), H_2_O_2_, 4-Nitroquinoline N-oxide (4-NQO) (Sigma, N8141), menadione (MedChemExpress), and N-acetyl-cysteine (NAC) (Sigma, A7250).

### Immunoblot, immunoprecipitation, nonreducing protein immunoblot experiment, and Phos-tag gels

The cell lysate samples were obtained in lysis buffer with protease inhibitor cocktail (Roche, 4693132001), and the immunoblot experiment was performed as previously described. Briefly, the proteins in the samples were first separated using an appropriate SDS-PAGE gel by electrophoresis and then transferred onto a polyvinylidene difluoride (PVDF) membrane (Merck Millipore, IPVH00010). The proteins on the membrane were next subjected to immunoblot using primary antibodies for 4 h and horseradish peroxidase-conjugated goat anti-mouse or anti-rabbit antibodies (Jackson ImmunoResearch Laboratories) for 2 h. The immunoblot experiment results were finally visualized using an ECL detection kit (Thermo Fisher Scientific, 34080). In immunoprecipitation analysis, the harvested cell lysates were centrifuged (12,000 *g* for 15 min at 4 °C). The indicated proteins in supernatants were purified by Protein G Magnetic Beads (Invitrogen, 10004D), which were preincubated with corresponding antibodies. Finally, the immunoblot analysis was used to visualize protein binding results of immunoprecipitation. To analyze the high molecular weight (HMW) oligomer of tau, we used a non-reducing protein immunoblot experiment, in which the protein samples do not contain reducing agents. The Mn^2+^-Phos bind SDS-PAGE (Phos-tag gels) have been described previously (58). Briefly, the Phos-tag gels, which are widely used to analyze the protein phosphorylation level, contain 65 μM MnCl_2_ and 25 μM Phos-tag (APExBIO, F4002), and the Phos-tag gels should be washed to remove excess Mn^2+^ using 10 mM EDTA before the immunoblot experiment.

The following primary antibodies were used: anti-GFP antibody (Santa Cruz, sc-9996), anti-SC-35 antibody (Abclonal, A3635), anti-mCherry antibody (abbkine, a02080), anti-tau antibody (abcam, ab80579), and anti-GAPDH antibody (Proteintech, 60004-4-Ig).

### Immunofluorescence assay, dye staining assays, and live cell imaging

Immunofluorescence staining and live cell imaging experiments were performed as we previously described (56, 59, 60). In an immunofluorescence staining experiment, the cultured cells were first fixed using 4% paraformaldehyde and permeabilized using 0.1% Triton X-100. Then the cells were subjected to immunofluorescence staining using the indicated primary antibodies and corresponding fluorescent secondary antibodies. Finally, the stained cells were visualized using a confocal microscope. In live cell imaging, the cells were cultured in a specific incubator (37 °C, 5% CO_2_, and a humidified atmosphere) and were imaged using a confocal microscope.

The following antibodies and secondary antibodies were used: anti-SC-35 antibody (Abclonal, A3635), anti-MAP2 antibody (Proteintech, 17490-1-AP), anti-p-tau antibody (T231) (Cell Signaling Technology, 71429), anti-DCP1a antibody (ABclonal, A7376), anti-NONO antibody (Proteintech, 11058-1-AP), anti-Coilin antibody (Proteintech, 10967-1-AP), anti-SC-35 antibody (Sigma, S4045), Alexa Fluor 488 Goat anti-mouse IgG (H + L) (Proteintech, SA00013-1), Alexa Fluor 594 Goat anti-rabbit IgG (H + L) (Proteintech, SA00013-4), and Alexa Fluor 594 Goat anti-mouse IgG (H + L) (Proteintech, SA00013-3). The staining assays with dyes were performed as we previously described (55, 61), and the following dyes were used: propidium iodide (PI) (Beyotime, P4170), Hoechst (MedChemExpress), and Thioflavine S (ThS) (MedChemExpress, HY-D0912).

### Recovery after activation of the optogenetic model and fluorescence recovery after photobleaching (FRAP)

Recovery after activation of the optogenetic model and fluorescence recovery after photobleaching (FRAP) were performed as we previously described (55). The opto-tau protein expressed in cells was activated by blue light (488 nm wavelength), and then was imaged by 561 nm wavelength to observe the disassembly process of condensates. In the FRAP experiment, the condensates were photobleached by 100% power 488 nm light for 40 s, and then were sequentially imaged to observe their fluorescence recovery process. In FRAP result quantification, the fluorescent intensity of pre-photobleaching condensates was normalized to 1, and the fluorescent intensity of first photobleaching condensates was normalized to 0.

### Animal brain sample preparation and primary cortical neuron preparation

The preparation of brain homogenates from 3xTg-AD (Psen1^M146V^, APP^Swe^, and tau^P301L^) mice (Jackson Laboratory) and C57BL/6 mice were previously described (62). Briefly, the brains were first removed from mice, and tissue homogenate was prepared in ice-cold sterile PBS using a probe sonicator, followed by centrifugation for 15 min at 21,000 *g* to remove unwanted insoluble material and cellular debris. The protein content of supernatants was then determined by bicinchoninic acid (BCA) (Beyotime Biotechnology, ST2222), and supernatants were stored at −80 °C.

The preparation of primary mouse cortical neurons was performed as we previously described (59). Briefly, cortical neurons were separated from the cortex of C57BL/6 mouse embryos (embryonic day 17) and then were cultured in 90% neurobasal medium (Gibco, 21103049) and 10% FBS for 8 h. The medium was further replaced with supplemented neurobasal medium (without serum) for 7 to 9 days, and the neurons were finally used in the experiment and observed under a microscope.

### Statistical analysis

The schematic diagrams were drawn using Adobe Illustrator 2019 software, and the fluorescent pictures obtained from the microscope were processed using Nikon and ImageJ software. Cellular localization of nuclear speckle protein and Hoechst was determined by ImageJ software. The charts about the obtained data were performed by Prism 10.0 (GraphPad) software, and the *p*-values were displayed in figure legends.

## Data availability

All data described have been included in the manuscript.

## Supporting information

This article contains supporting information.

## Conflict of interest

The authors declare that they have no conflicts of interest with the contents of this article.
