# Supplementary material for: Oxidative modification alters tau condensation and promotes tau-associated cytoplasmic speckle formation
Source: J Biol Chem. 2026 Jul 24;302(9):113365. doi: 10.1016/j.jbc.2026.113365 (PMC13524881; doi:10.1016/j.jbc.2026.113365)
Supplement: Supplementary file 1 [file mmc1.pdf]

Supplemental Figure 1

A

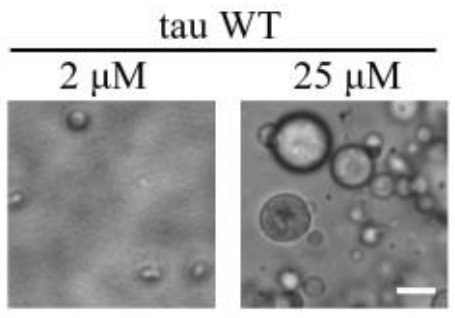

Figure S1. tau undergoes phase separation *in vitro*. (**A**) Phase separation of unlabeled tau (2 μM, 25 μM) with molecular crowding agent (10% PEG4000) and polyU (0.01 μg/μL) in 150 mM NaCl *in vitro*. Scale bar, 10 μm.

Supplemental Figure 2

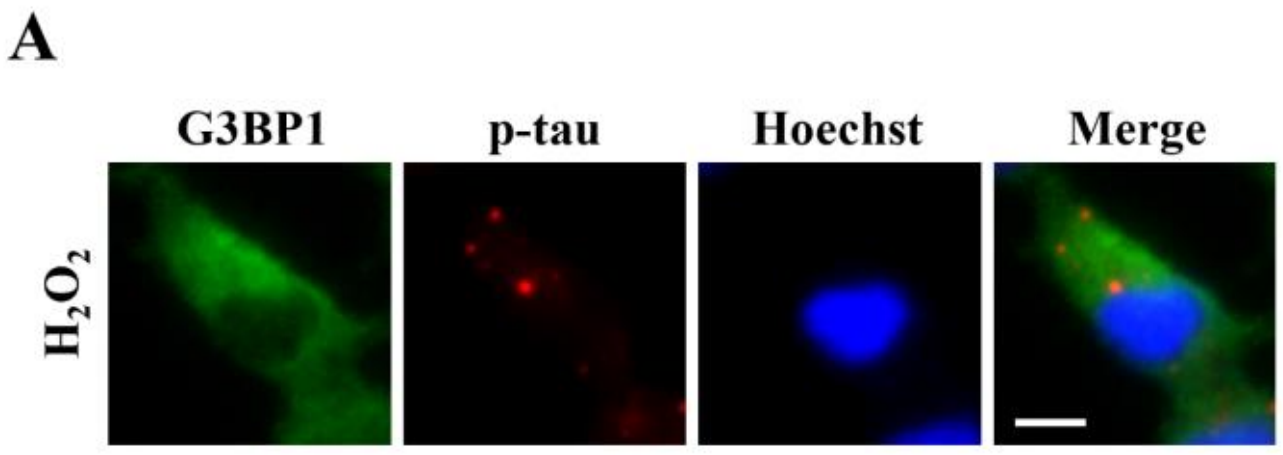

Figure S2. TACS differs from G3BP1-marked stress granule. (**A**) HEK 293 cells were treated with 200 μM H_2O_2 for 15 min, and then were fixed and stained with anti-G3BP1, anti-SC-35 (SRSF2) antibodies and Hoechst. Scale bar, 10 μm.
